# Toothbrush-Driven Handheld Droplet Generator for Digital LAMP and Rapid CFU Assays

**DOI:** 10.3390/bios16010030

**Published:** 2026-01-01

**Authors:** Xiaochen Lai, Yong Zhu, Mingpeng Yang, Xicheng Wang

**Affiliations:** 1 School of Artificial Intelligence, Nanjing University of Information Science and Technology, Nanjing 210044, China; 2 School of Automation, Nanjing University of Information Science and Technology, Nanjing 210044, China; 202312490055@nuist.edu.cn (Y.Z.); mpyang@nuist.edu.cn (M.Y.); 202312490022@nuist.edu.cn (X.W.)

**Keywords:** handheld droplet generator, digital assay, sample dispersion

## Abstract

Droplet microfluidics enables high-throughput, compartmentalized reactions using minimal reagent volumes, but most implementations rely on precision-fabricated chips and external pumping systems that limit portability and accessibility. Here, we present a handheld vibrational droplet generator that repurposes a consumer electric toothbrush and a modified disposable pipette tip to produce nearly monodisperse water-in-oil droplets without microfluidic channels or syringe pumps. The device is powered by the toothbrush’s built-in motor and controlled by a simple 3D-printed adapter and adjustable counterweight that tune the vibration amplitude transmitted to the pipette tip. By varying the aperture of the pipette tip, droplets with diameters from ~100–300 µm were generated at rates of ~100 droplets s^−1^. Image analysis revealed narrow size distributions with coefficients of variation below 5% in typical operating conditions. We further demonstrate proof-of-concept applications in digital loop-mediated isothermal amplification (LAMP) and microbiological colony-forming unit (CFU) assays. A commercial feline parvovirus (FPV) kit manufactured by Beyotime Biotechnology Co., Ltd. (Shanghai, China), three template concentrations yielded emulsified reaction droplets that remained stable at 65 °C for 45 min and produced distinct fractions of fluorescent-positive droplets, allowing estimation of template concentration via a Poisson model. In a second set of experiments, the device was used as a droplet-based spreader to dispense diluted *Escherichia coli* suspensions onto LB agar plates, achieving uniform colony distributions across the plate at different dilution factors. The proposed handheld vibrational generator is inexpensive, easy to assemble from off-the-shelf components, and minimizes dead volume and cross-contamination because only the pipette tip contacts the sample. Although the current prototype still exhibits device-to-device variability and moving droplets in open containers complicate real-time imaging, these results indicate that toothbrush-based vibrational actuation can provide a practical and scalable route toward “lab-in-hand” droplet assays in resource-limited or educational settings.

## 1. Introduction

Droplet-based microfluidics has become a central platform for miniaturized biochemical assays and high-throughput experimentation. By compartmentalizing reactions into monodisperse, picolitre–nanoliter droplets dispersed in an immiscible carrier fluid, droplet systems dramatically reduce reagent consumption, improve reaction kinetics and enable large numbers of parallel experiments that would be impractical in conventional microtubes or microplates [1,2,3]. Early reviews by Teh et al. and Theberge et al. summarized how monodisperse droplets can serve as isolated microreactors for synthesis, enzyme screening and single-cell studies, and highlighted their potential to transform analytical chemistry and biology [4,5]. Subsequent work has expanded these ideas across pharmaceutical development, microbiology, single-cell omics and synthetic biology, establishing droplet microfluidics as an indispensable tool in contemporary life science research [6,7,8].

Digital nucleic-acid assays are among the most widely adopted applications of droplet technology. In digital PCR (dPCR) and droplet digital PCR (ddPCR), a bulk sample is partitioned into thousands to millions of nanoliter or sub-nanoliter compartments, each behaving as a yes/no microreaction whose positivity reflects the presence of one or more target molecules [9]. Counting fluorescent-positive droplets and invoking Poisson statistics enables absolute quantification of DNA or RNA copies without external calibration curves [10]. Over the last decade, a rich ecosystem of droplet- and chamber-based dPCR devices has emerged, including highly integrated chips for oncology and infectious disease testing [11]. More recently, loop-mediated isothermal amplification (LAMP) has been combined with droplet partitioning to create digital LAMP (dLAMP) platforms [12], which leverage the rapid, single-temperature amplification and inhibitor tolerance of LAMP while preserving the absolute quantification advantages of digital read-out [13,14,15]. These dLAMP approaches span droplet array chips, membrane-based devices and fully integrated cartridges for continuous partitioning, incubation and fluorescence detection [16].

Parallel to nucleic-acid detection, droplet microfluidics has also advanced microbiology. Encapsulating individual bacteria in droplets allows digital colony-forming unit (CFU) counting and single-cell phenotyping over wide dynamic ranges [17]. Scheler and co-workers optimized a droplet digital CFU (ddCFU) assay that quantifies bacterial loads over six orders of magnitude by fitting the fraction of growth-positive droplets to a Poisson model [15]. Kao et al. later implemented a gravity-driven droplet platform for digital enumeration of bacteria and antibiotic susceptibility testing, underscoring how digital microbiology can deliver precise counts and minimum inhibitory concentration measurements in a single workflow [18]. More recently, microfluidic “digital plating” platforms have begun to bridge droplet-based assays with classical agar-plate microbiology, enabling controlled deposition of droplets onto solid media for robust CFU analysis and colony isolation [19,20,21]. These developments collectively demonstrate that droplet techniques can support both molecular diagnostics and culture-based microbiology within unified frameworks.

The mechanisms and efficiency of droplet or bubble formation in microchannels have been studied in depth, and a broad set of strategies has been developed to improve breakup stability, throughput, and size control [22,23]. Representative passive approaches rely on interfacial shear within canonical geometries such as T-junctions, flow-focusing, and co-flow configurations, where carefully designed channel constraints promote reproducible pinch-off [24]. For example, Shen demonstrated that a simple geometric modification—introducing a glass capillary into a microchannel—can create local confinement that enhances droplet separation and controllability, yielding a more stable droplet stream [25]. Beyond purely passive designs, active or hybrid methods further expand the controllable parameter space: a Physics of Fluids study showed that applying an electric field to flow-focusing or T-junction microchannels can reshape the interface and alter the effective shear conditions, thereby increasing breakup efficiency and droplet generation frequency [26]. Meanwhile, work reported in the International Journal of Multiphase Flow introduced a redesigned T-junction capable of alternating one-to-one droplet formation [27], where precise tuning of flow rates and geometry enabled sequential droplet production from different phases, highlighting the strong coupling between channel design, hydrodynamics, and multiphase partitioning.

Despite these advances, conventional droplet microfluidic implementations remain technically demanding in practice. Most systems still depend on microfabricated chips incorporating flow-focusing or T-junction structures, driven by syringe or pressure pumps and supported by ancillary valves, tubing, and control electronics [28]. Achieving stable, monodisperse droplet generation typically requires tight regulation of flow rates, surfactant formulation, and surface wetting conditions; even modest fluctuations in pump performance or gradual channel fouling can rapidly compromise droplet uniformity [29]. As a result, the operational complexity and infrastructure requirements continue to limit accessibility to laboratories with microfabrication capabilities and experienced users, and they hinder adoption in low-resource or point-of-care scenarios. This barrier is particularly evident in microbiology and teaching laboratories, where the assays themselves may be conceptually straightforward, yet the need for specialized chips and external pumping hardware remains a major obstacle to broader dissemination [24].

To address these issues, considerable effort has gone into simplifying droplet hardware and flow control. Several groups have developed pump-free or hand-powered droplet generators based on negative pressure, gravity, spring-driven syringes or vacuum-assisted actuation [30]. For example, Chen et al. reported a hand-held, power-free microfluidic device that uses a pre-evacuated syringe to drive flow through a droplet-generation chip, producing monodisperse sub-nanoliter droplets with coefficients of variation below 8% [31]. Han et al. designed a wind-up mechanical pump that maintains nearly constant syringe flow without electricity, while Fajrial et al. introduced a frugal gravity-assisted pump built from commodity components [32]. Other minimal droplet generators integrate microchannel networks with small negative-pressure sources or laptop-controlled valves to reduce footprint and setup complexity [33]. These approaches substantially lower system cost and improve portability, yet they still depend on microfabricated channels and chip-to-world connections that can be prone to leakage, clogging and sample loss.

Droplet generation strategies in microfluidics span a broad spectrum, from chip-free handheld systems to highly integrated, actively controlled lab-on-a-chip platforms, with substantial differences in actuation principles, integration level, and required infrastructure. On the chip-free side, droplets can be generated directly from simple capillaries or pipette tips by applying acoustic, mechanical, or centrifugal excitation to destabilize a liquid jet and drive controlled breakup without the need for enclosed microchannels. Representative examples include vibrating sharp-tip capillary systems, in which piezoelectric actuation induces acoustic streaming and produces droplets with finely tunable diameters and high throughput—features that are particularly attractive for quantitative workflows such as digital PCR—albeit at the cost of bespoke actuators and careful alignment between the vibration source and the nozzle geometry [34]. Similarly, acoustically activated nozzles can generate and dispense microdroplets via acoustic streaming at the capillary interface, enabling excellent size control and rates up to ~2000 droplets/s, but typically requiring customized transducers and control electronics to ensure reproducible operation [35]. These chip-free approaches reduce fabrication burden, minimize dead volume in tubing, and simplify cleaning, yet the dependence on specialized hardware and alignment procedures can still limit adoption outside specialist laboratories.

At the opposite end of the spectrum are integrated, chip-based active droplet generators, such as microvalve platforms, which can precisely coordinate droplet generation, splitting, and merging on a single microfluidic chip to support complex fluidic sequencing and high-throughput screening. For example, Agnihotri et al. demonstrated a multi-site microvalve system that achieves programmable droplet operations through on-chip valves, but this level of functionality generally relies on microfabrication, external pressure controllers, and sophisticated control hardware [36].

The toothbrush-driven droplet generator presented here occupies a practical niche between these extremes in terms of actuation simplicity, infrastructure requirements, and achievable performance. By repurposing a consumer-grade toothbrush motor and pairing it with a simple fluidic interface, it enables chip-free droplet generation with high accessibility and minimal dependence on external equipment. Unlike vibrating-capillary or acoustofluidic systems, it does not require bespoke electronics or precise actuator–nozzle alignment; unlike microvalve-based platforms, it eliminates microfabricated chips and external pumping hardware. The trade-off is that throughput and droplet size uniformity are presently more modest than those achieved by specialized high-performance platforms. Nevertheless, the ultra-low cost, ease of use, and integration of actuation and handling into a single handheld unit make this approach well suited for resource-limited settings, rapid field deployment, and point-of-care digital workflows where simplicity and accessibility are prioritized.

In parallel with these engineering efforts, there is growing interest in “frugal science” and repurposed consumer hardware for laboratory applications, where low-cost devices based on toys, optical drives or smartphones are adapted as centrifuges, microscopes and analyzers for resource-limited settings [37]. In the context of droplet workflows, however, most existing frugal devices still operate upstream or downstream of chip-based generators rather than replacing the generator itself.

Here we propose a different approach: a handheld vibrational droplet generator that repurposes a commercial electric toothbrush as both actuator and handle. A disposable, heat-modified pipette tip is mounted on a compact 3D-printed adapter attached to the toothbrush shaft. By tuning a small counterweight on the shaft, the strong, well-defined vibrations produced by the toothbrush are converted into a controlled lateral oscillation at the nozzle, yielding a single stable jet that breaks into nearly monodisperse water-in-oil droplets at rates of ~100 droplets s^−1^. In contrast to conventional microfluidic generators, our system is entirely chip-free and pump-free: there are no microchannels, valves or external pressure sources, and the only wetted component is the disposable pipette tip. This configuration minimizes dead volume, avoids cross-contamination between assays, and confines all fabrication to 3D-printed parts that can be readily reproduced.

We further show that droplets produced by this toothbrush-based generator are compatible with both nucleic-acid amplification and microbiological culture. As proof of concept, we perform droplet digital LAMP using a feline parvovirus detection kit and demonstrate that the fraction of fluorescent-positive droplets decreases systematically with template dilution, consistent with Poisson statistics. In a complementary application, we use the same device as a droplet-based spreader to distribute diluted *Escherichia coli* suspensions over LB agar plates, achieving uniform colony distributions that are suitable for rapid CFU estimation. Together, these results suggest that a simple, repurposed toothbrush can support “lab-in-hand” droplet workflows that bridge digital molecular assays and classical plate microbiology, while avoiding the fabrication and operational complexity of conventional microfluidic platforms.

## 2. Materials and Methods

### 2.1. Handheld Vibrational Droplet Generator

#### 2.1.1. Electric Toothbrush Modification

**Figure 1:**
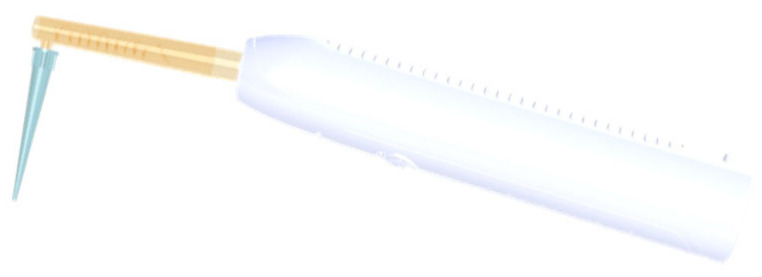
Overview of the droplet system, featuring an electric toothbrush with a custom 3D-printed device and a modified pipette tip for droplet formation.

A rechargeable sonic electric toothbrush (Philips Sonicare HX6210 Koninklijke Philips N.V., Amsterdam, The Netherlands) served as the vibration source. The original brush head was removed and replaced by a custom 3D-printed adapter (approximately 68 mm in length and about 10 mm in diameter) that mechanically couples the toothbrush shaft to a disposable pipette tip (Figure 1). The adapter contains an internal channel with a diameter of 1.2 mm and converts the rotary motion of the internal motor into lateral vibrations at the distal end where the tip is mounted.

To overcome the limited built-in amplitude settings of the toothbrush, we implemented a simple, prototype counterweight strategy. A roughly fabricated counterweight was initially attached to the infusion set to perturb the vibration response, and these preliminary tests confirmed that introducing a counterweight measurably changes the vibration dynamics and, consequently, droplet formation behavior. We then adopted a compact 3D-printed counterweight (~0.5 g) mounted eccentrically on the moving shaft. By varying the counterweight mass and its radial offset from the rotation axis, we were able to fine-tune the effective vibration amplitude transmitted to the pipette tip, enabling operation within a narrow regime where a single, stable jet forms and monodisperse droplets are produced. At this stage, we have not yet quantitatively mapped counterweight mass/position to droplet output metrics (e.g., droplet production rate, size distribution, and jetting stability); this will be addressed in future work through systematic parametric studies, potentially complemented by schematic illustrations to clarify the tuning mechanism. The assembled device is approximately 15 cm in length, weighs ~8 g, and is powered solely by the toothbrush’s internal battery, allowing fully cord-free operation.

#### 2.1.2. Pipette-Tip Nozzle Fabrication

**Figure 2:**
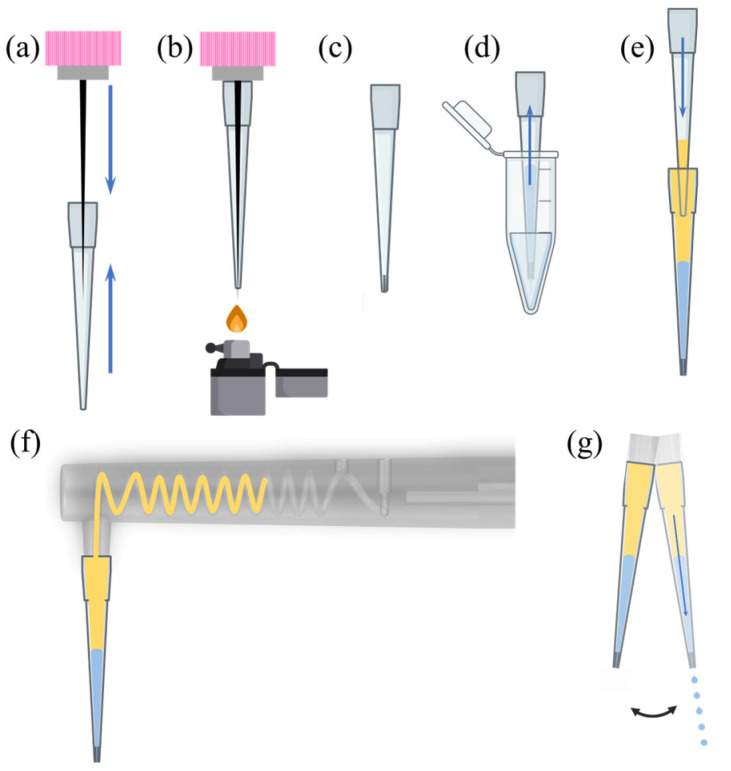
Fabrication of the custom pipette tip and droplet generation. (**a**) Insertion of tungsten microneedles into a pipette tip, with an adjustable spacer (grey) to vary the aperture size. (**b**) Heat treatment of the pipette tip using a lighter. (**c**) Removal of the microneedles, leaving a custom pipette tip. (**d**) Attachment of the pipette tip to a micropipette, drawing the aqueous phase. (**e**) Addition of the oil phase above aqueous phase. (**f**) Installation of the pipette tip onto the 3D-printed device preloaded with oil phase. The yellow region indicates the oil used to assist droplet generation (ABIL EM 180/isopropyl palmitate emulsion, mentioned in the text below). (**g**) Activation of the electric toothbrush to generate droplets via shear and centrifugal forces.

Standard 20 µL polypropylene pipette tips (length ~5 cm) were used as both sample reservoir and droplet nozzle. To obtain well-defined small apertures, we inserted a tungsten microneedle into the tip, heated the plastic locally with a lighter or heat gun, and then allowed it to cool before removing the needle (Figure 2a–c). This produced a tapered opening with a diameter in the range 80–300 µm depending on the needle size and heating time.

In our experiments, tungsten needles were used to fabricate the nozzle apertures, with a tip diameter of approximately 0.01 mm. The use of a tungsten needle was intended to ensure that, during the forming process, the aperture would not fuse or collapse, thereby maintaining the integrity and independence of each opening. Although the tungsten needle enabled the reliable fabrication of small apertures, the aperture size could not be controlled with perfect precision due to practical limitations of the heating/reshaping process. As a result, while this approach can produce a large number of small-aperture nozzles with reasonable effort and is suitable for droplet generation, some variability in aperture size and consistency is expected.

To improve droplet size stability and reproducibility, we adjusted and optimized the tungsten-needle-based fabrication procedure multiple times according to the requirements of droplet generation. In practice, we primarily used nozzles with an aperture of ~90 µm to meet the experimental needs, and we further evaluated droplet generation efficiency and stability using this aperture size. This approach allowed us to maintain adequate droplet throughput while minimizing variability in the nozzle aperture.

For other laboratories aiming to reproduce this device, we recommend using tungsten needles with a tip diameter of ~0.01 mm and performing appropriate heat-forming to obtain apertures with the desired size and shape for droplet generation. If tighter control of the aperture size is required, the aperture can be fine-tuned by combining microscopic inspection with additional measurement tools.

Different tip apertures were prepared for different experiments. For droplet size characterization and LAMP assays we used apertures of ~80–110 µm to generate smaller droplets. For some demonstration images, larger apertures (~180–300 µm) were used to highlight the relationship between nozzle size and droplet diameter.

Immediately before use, the modified tip was mounted on a manual micropipette, filled with aqueous sample, topped with oil (for in-oil emulsions), and then transferred to the 3D-printed adapter on the toothbrush (Figure 2d–g). Because the sample contacts only the disposable tip, there is no need to clean the device between assays.

### 2.2. Continuous Phase Oils

Two oil systems were used in this work:

- Mineral oil with Span 80 (2 wt%) was used in early experiments to screen general droplet-generation behavior and stability at room temperature. Mineral oil has moderate viscosity and, combined with the nonionic surfactant Span 80, supports stable water-in-oil droplets for several hours.
- ABIL EM 180/isopropyl palmitate emulsion (7:93 wt%) was used for LAMP assays. This biocompatible oil–surfactant mixture has been widely used for encapsulating biochemical reactions and provides robust droplet stability at 60–65 °C.

For all experiments, 5–10 mL of the continuous phase was placed into a 75 mm diameter polystyrene Petri dish. The pipette tip was positioned close to the oil surface so that droplets detached from the tip and immediately entered the oil.

### 2.3. LAMP Reagents and Droplet LAMP Protocol

A commercial feline parvovirus (FPV) LAMP fluorescence detection kit (Beyotime Biotechnology, Shanghai, China) was used as the nucleic-acid assay. The kit detects FPV DNA by loop-mediated isothermal amplification with fluorescence read-out and includes dUTP and UDG to minimize carry-over contamination from previous LAMP products. Among them, Bst DNA polymerase is a DNA polymerase with strong strand-displacement activity; the Positive Control (FPV DNA) is a known positive control; and UDG is mainly used to eliminate carry-over contamination arising from amplification products during PCR/LAMP reactions.

The standard 20 µL reaction mixture was prepared according to the manufacturer’s instructions (Table 1). For the “normal” concentration, the Positive Control provided with the kit was used at the recommended stock concentration; for diluted samples, the Positive Control was serially diluted 5-fold and 25-fold with nuclease-free water before adding to the reaction.

For each concentration, the reaction mixture was loaded into a modified pipette tip, followed by an appropriate volume of ABIL EM180/isopropyl palmitate oil phase. The tip was then mounted on the toothbrush device and actuated to generate droplets directly into a 75 mm Petri dish pre-filled with the same oil phase. Under typical conditions, ~100 droplets were produced per second, with diameters of 80–110 µm.

After droplet generation, dishes were placed in a temperature-controlled oven at 65 °C for 45 min to perform isothermal amplification. Following incubation, droplet fluorescence images were acquired using an epifluorescence microscope (FITC channel) with exposure time 250 ms and magnification 4×.

### 2.4. Digital LAMP Image Analysis and Poisson Model

Fluorescence images were analyzed in MATLAB (Version R2024b). Briefly, images were first smoothed and background-corrected, then converted to binary images using a size filter of 60–110 µm and an intensity threshold of 100. Circular Hough transform or connected-component analysis was used to identify individual droplets. For each droplet, mean fluorescence intensity was calculated.

A global intensity threshold was chosen to classify droplets as positive or negative. For each sample, the total number of droplets and number of positive droplets were recorded. Let n be the total number of droplets, h the number of positives and b=n−h the number of negatives; the observed positive fraction is

$$p=\frac{h}{n}.$$

Assuming that template molecules are randomly distributed among droplets according to a Poisson process, the probability that a droplet contains at least one template molecule is

$$p=1-e^{-\lambda },$$

where λ is the mean copy number per droplet. Thus,

$$\lambda =-\ln\left(1-p\right).$$

To convert λ into an estimated template concentration c (copies mL^−1^), we approximate droplets as spheres with radius r (mean radius estimated from diameter measurements), giving a droplet volume $v=4\pi r^{3}/3$. If the initial sample volume partitioned into droplets is V, then

$$c=\frac{\lambda }{v}.$$

Because the present work aims only to demonstrate feasibility rather than provide a fully calibrated quantitative assay, we emphasize relative concentration comparisons: The relationship between λ and the known dilution factor x was examined by plotting λ versus ${log}_{10}(x)$ and fitting a linear regression.

### 2.5. E. coli Culture and Plate Dispersion

#### 2.5.1. Bacterial Strain and Growth Conditions

A laboratory *Escherichia coli* strain transformed with a Plac-EGFP plasmid was used as the model microorganism. Cells were grown overnight in LB broth at 37 °C with shaking at 200 rpm. LB medium (broth and agar) was prepared using standard recipes containing tryptone, yeast extract and NaCl.

#### 2.5.2. Preparation of Dilution Series

The overnight culture was diluted in sterile nuclease-free water to obtain three concentrations: undiluted (1×), 10-fold dilution (10×) and 100-fold dilution (100×). Dilutions were performed in autoclaved tubes using sterile pipette tips, with vigorous mixing to ensure homogeneity. Actual CFU concentrations were later estimated from colony counts (Section 3.4).

#### 2.5.3. Plate Inoculation Using the Droplet Generator

For each dilution, 50 µL of bacterial suspension was loaded into a modified pipette tip. LB agar plates (90 mm diameter) were pre-warmed to room temperature. The droplet generator was positioned above the plate center and actuated for 8 s, producing a fine spray of droplets that spread from the center towards the periphery. Three replicate plates were used per dilution (*n* = 3), each prepared in an independent run.

After inoculation, plates were inverted and incubated at 37 °C for 24 h. Colonies were then imaged using a gel-documentation system or DSLR camera under ambient light and, when EGFP fluorescence was required, under blue excitation.

### 2.6. Microscopy and Droplet Size Analysis

Bright-field images of emulsified droplets were acquired with an inverted microscope at 4× magnification. Droplet diameters were measured using MATLAB (Version R2024b): images were binarized, objects with areas corresponding to diameters of 60–400 µm were selected, and equivalent circular diameters were computed. Histograms were constructed from at least 300 droplets per condition, and mean diameter and standard deviation were reported.

## 3. Results

### 3.1. Vibration Tuning and Jet Formation Regimes

Without counterweight tuning, the toothbrush’s built-in amplitude levels provide only coarse control over the vibration transmitted to the pipette tip. At the lowest setting, the aqueous phase forms a large pendant droplet at the nozzle that detaches only occasionally, producing millimeter-scale droplets and very low throughput. When the amplitude is suddenly increased to the highest setting, liquid is expelled violently in multiple directions, leading to unstable jets and a wide spread of droplet sizes.

**Figure 3:**
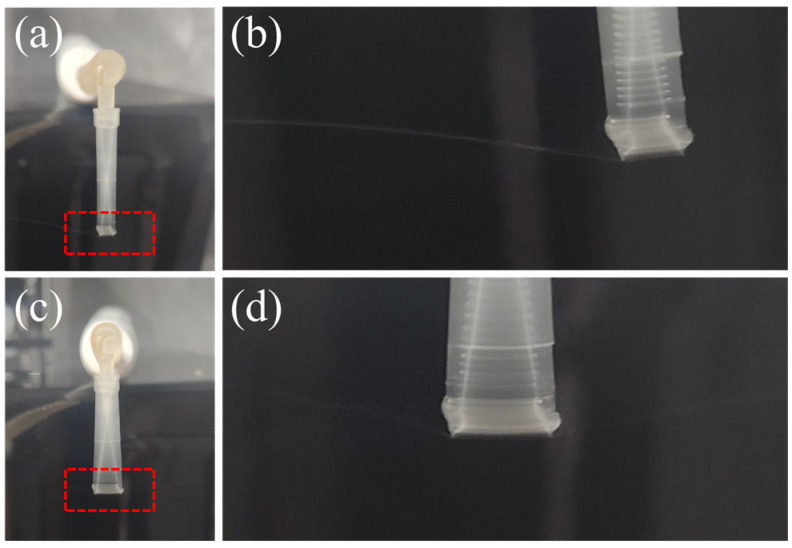
Droplet generation at varying vibration amplitudes. (**a**) Stable single-jet droplet formation with moderate vibration amplitude. (**b**) Zoomed-in view of (**a**). (**c**) Multiple jets formed under higher vibration amplitude, leading to droplets of varying sizes in multiple jets. (**d**) Zoomed-in view of (**c**), showing the multiple jets in different directions.

Adding an adjustable counterweight to the toothbrush shaft effectively smooths these transitions. By gradually increasing the counterweight mass and shifting its position, we attenuated the amplitude to a regime where the pipette tip undergoes a controlled lateral oscillation. In this window, a single, narrow jet emerges from the nozzle and breaks into droplets at a nearly constant distance from the tip (Figure 3a,b). If the amplitude is further increased, the jet splits into multiple branches (Figure 3c,d), again broadening the droplet size distribution.

**Figure 4:**
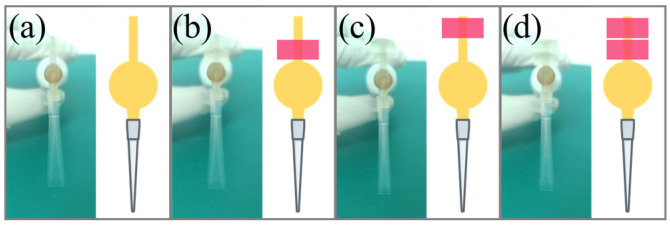
Fine-tuning the vibration amplitude using a counterweight to achieve stable droplet formation. (**a**) Before adjustment. (**b**–**d**) After gradually adding and repositioning the counterweight, the moment of inertia of the device is increased, and the amplitude of vibration can be thus attenuated. Such finetuning allowing on-demand droplet generation by providing a single jet stream.

Thus, the counterweight transforms the toothbrush from a two-level actuator into a tunable vibrational source and is essential for robust single-jet operation across different tip geometries, as depicted in Figure 4.

### 3.2. Droplet Size Control and Monodispersity

**Figure 5:**
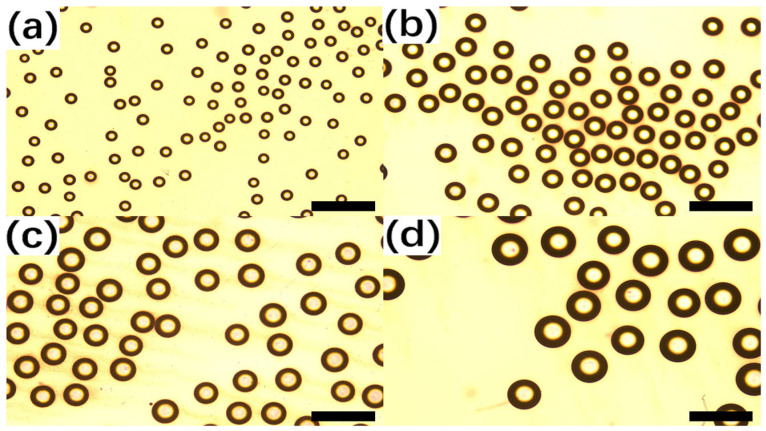
Generation of droplets with varying size, varying from 100 μm (**a**), 180 μm (**b**), 220 μm (**c**) to 300 μm (**d**). The variation in droplet size is directly related to the aperture size of the pipette tip used. Scalebar: 500 μm.

**Figure 6:**
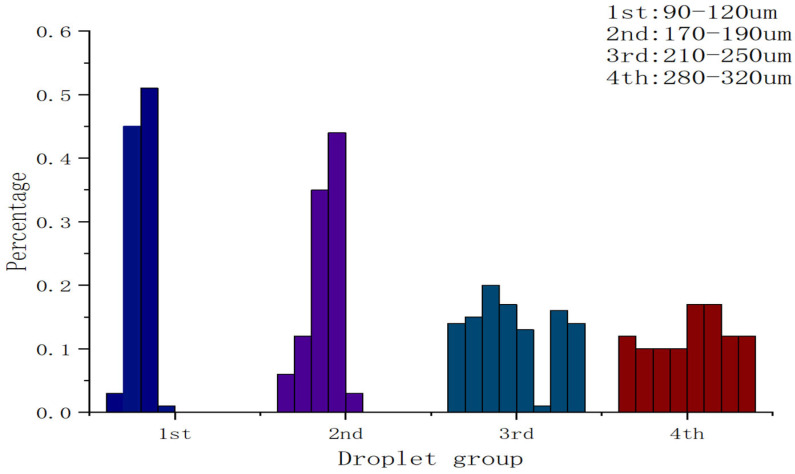
Droplet size distribution histogram. Different colors represent droplets generated by different aperture.

Figure 5 shows representative droplet fields generated using four pipette tips with different nozzle apertures. As expected from capillary-breakup physics, droplet diameter increases with nozzle size: tips with ~90, 180, 220 and 300 µm apertures produced droplets centered around approximately 100, 180, 220 and 300 µm, respectively. Histograms constructed from >300 droplets per condition (Figure 5) reveal narrow distributions with coefficients of variation of (3–5%) for the optimized operating point. Meanwhile, for each nozzle aperture, droplets with different size distributions were generated, as analyzed in Figure 6.

In the droplet-generation experiments, the nozzle aperture was approximately 90 µm and enabled stable droplet production. Based on multiple experimental runs, the average generation rate under the specified operating conditions was ~106 droplets per second. Each run could sustain stable jetting for tens of seconds; therefore, the total number of droplets produced in a single experiment typically exceeded 1000. The droplet diameter was mainly distributed in the range of 90–110 µm. Supplementary Video S1 demonstrates the droplet generation process and shows droplets falling into the oil phase.

To evaluate how the droplet count varied with operation time, we used the same device with this nozzle and performed droplet-generation runs for 5, 10, 15, and 20 s. As shown in Table 2. Droplets were then imaged and counted in selected local fields of view under a microscope, and the total droplet number was estimated accordingly. Owing to the current limitations in imaging and counting, the reported droplet counts should be regarded as approximate; the actual number may vary with experimental conditions and fluctuations in device performance.

Across four runs (5–20 s), the estimated droplet generation rate ranged from ~85 to ~115 droplets s^−1^ (mean ≈ 106 droplets s^−1^).

**Figure 7:**
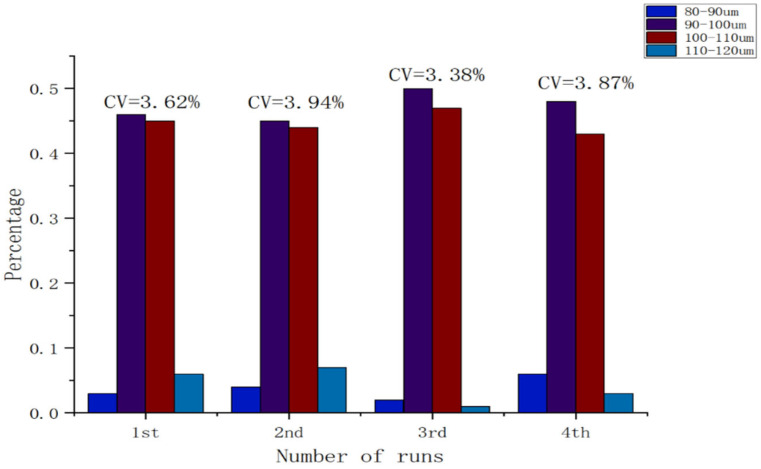
Droplet size distribution (count) generated with the ~90 µm nozzle aperture.

Because droplets generated using the ~90 µm nozzle aperture were the most stable, we conducted multiple experiments using different devices fitted with nozzles of the same nominal aperture to examine variations in droplet size. In all experiments, droplets were generated in a Petri dish for a fixed duration of 5 s, and regions with relatively high droplet density were selected for microscopic observation. Across four experimental runs, the droplet diameter coefficient of variation (CV) was 3.62%, 3.94%, 3.38%, and 3.87%, respectively. The mean CV was approximately 3.70%, indicating good monodispersity (low size polydispersity) and high run-to-run consistency in droplet size. It should be noted that, due to differences in experimental conditions and device performance, the total number of droplets varied across experiments. The size distribution is summarized in Figure 7.

We also evaluated droplet stability at room temperature and under LAMP incubation conditions. At room temperature, no coalescence was observed over at least 2 h, and mean diameter decreased by less than 5%, consistent with minor evaporation or slight oil uptake. When emulsions were heated to 65 °C for 45 min in the ABIL EM180/isopropyl palmitate formulation, droplet counts and diameters remained unchanged within the measurement uncertainty, confirming that the droplets withstand isothermal reaction conditions.

Given the simplicity of the device and manual tip fabrication, this level of monodispersity is sufficient for many digital assay and encapsulation tasks.

### 3.3. Proof-of-Concept Droplet Digital LAMP

**Figure 8:**
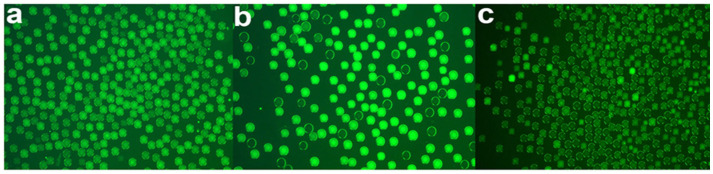
Droplet digital LAMP of feline parvovirus (FPV) using toothbrush-generated emulsions. Fluorescence images of water-in-oil droplets after LAMP amplification for (**a**) undiluted positive control, (**b**) 5-fold diluted template and (**c**) 25-fold diluted template. Bright droplets correspond to LAMP-positive partitions. The fraction of positive droplets decreases systematically with dilution, consistent with Poisson-limited digital quantification and confirming the compatibility of the handheld droplet generator with isothermal nucleic-acid assays.

We next evaluated the compatibility of the handheld droplet generator with nucleic-acid amplification by performing droplet-based digital LAMP using the feline parvovirus kit. Emulsified reaction droplets at three template concentrations (normal, 5-fold dilution, 25-fold dilution) were incubated at 65 °C for 45 min and imaged under fluorescence (Figure 8a–c).

Even without quantitative analysis, visual inspection clearly shows a stepwise decrease in the fraction of fluorescence-positive (bright) droplets with increasing template dilution: at the undiluted condition, nearly all droplets are positive; at 5× dilution, the number of positive droplets is markedly reduced; and at 25× dilution, only sparse positive droplets are observed (Figure 8a–c). We then applied the image-analysis workflow described in Section 2.4 to automatically segment droplets, extract their contours, and perform counting to obtain quantitative readouts.

To further assess reproducibility, we performed multiple independent replicate experiments for each template concentration under identical operating conditions, including independent droplet generation and reaction preparation. For each replicate, the same analysis pipeline was used to extract the total droplet count n, the number of positive droplets h, and the positive fraction *p* = *h*/*n*, from which the mean copy number per droplet was estimated using the Poisson model, *λ* = −*ln*(1 − *p*). Table 3 summarizes the total droplet counts and positive fractions across conditions.

Based on these data, using λ=−ln(1−p), the estimated mean copy numbers per droplet for Batch 1 at the three concentrations were approximately 5.2, 1.56, and 0.0806, corresponding to relative copy numbers of 1, 0.2, and 0.04, respectively. Fitting a linear model of λ vs. the logarithm of the relative concentration x yielded

$$\lambda =4.87x+0.29(R^{2}=0.98),$$

indicating a good correlation between measured mean copy number and expected dilution factor over the explored range.

Overall, *λ* decreased monotonically with increasing dilution, with acceptable inter-run variability, further supporting the compatibility of droplets generated by the handheld device with digital LAMP and the reproducibility of the readout.

We emphasize that this experiment is intentionally limited in scope: only one droplet size and three concentrations were tested, with a single batch per condition. The goal here is not to fully validate a quantitative FPV assay but to demonstrate that (i) droplets generated by the handheld device are compatible with isothermal amplification chemistry, (ii) the droplets remain stable under incubation, and (iii) differences in template concentration can be resolved via differences in the fraction of positive droplets.

A more comprehensive analytical characterization, including limit-of-detection, linear dynamic range, inter-run variability and comparison with benchtop droplet systems, is left for future work.

### 3.4. E. coli Plate Dispersion for CFU Counting

**Figure 9:**
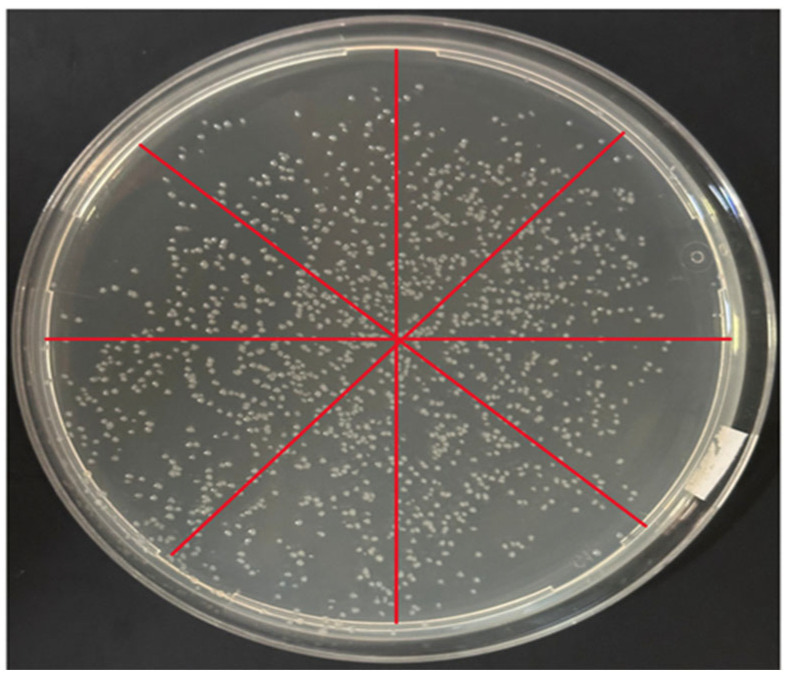
We evaluated the spatial uniformity of droplet distribution on the plate using two complementary approaches: (i) subdividing the plate into multiple sectors and concentric zones, followed by visual inspection and statistical comparison of the number of droplets (or colonies resulting from droplet inoculation) in each region; and (ii) counting droplets in representative microscopic fields of view and comparing counts across different regions to determine whether local clustering or droplet-free areas were present, thereby assessing distribution uniformity.

To assess the utility of the droplet generator for microbiological applications, we used it to distribute *E. coli* suspensions onto LB agar plates. Figure 9 shows representative bright-field images for the three tested concentrations (1×, 10×, 100×). At the highest concentration, colonies are numerous and relatively close to each other; at 10× dilution, colony density is reduced and individual colonies are easily distinguished; at 100× dilution, colonies are sparse but evenly scattered across the plate.

Across repeated inoculation runs, we adjusted the swaying motion to achieve uniform coverage. The sector-based analysis was performed on representative plates to quantify spatial uniformity, and Table 4 summarizes colony counts from triplicate plates per dilution. To quantitatively characterize the spatial distribution of colonies, we employed a sector-based partitioning method in preliminary CFU plate assays: each agar plate was divided into 4, 6, 8, or 16 radial sectors of equal angle, and an appropriate partitioning scale (8 divisions) was selected based on the overall colony coverage and density (Figure 9). Colony counts were then obtained for each sector to characterize the plate-wide spatial distribution, yielding values of 231, 233, 235, 237, 247, 249, 251, and 253, respectively.

To further quantify distribution uniformity, we calculated the coefficient of variation (CV = standard deviation/mean × 100%) of the sector-wise colony counts. Under the optimized operating conditions, the CV across sectors was approximately ~3.6%, indicating that the droplet-dispensing approach can produce a relatively uniform and reproducible colony distribution on agar plates.

Qualitatively, the droplet-based inoculation offers two benefits compared with manual streaking with an inoculation loop:

- Uniformity. Because the device emits many small droplets over the plate area in a short time, spatial distribution of bacteria is more uniform, and there are fewer regions of overcrowding or empty space.
- Labor saving and reproducibility. Inoculation requires only holding the device over the plate for a fixed time (8 s in our experiments), reducing operator-to-operator variability in streaking patterns and hands-on effort.

For full quantitative CFU analysis, colony counts should be obtained from multiple plates per dilution. As shown in Table 4. In the current proof-of-concept experiment, plates at each dilution level yielded approximately:

From these values, CFU mL^−1^ can be calculated in the usual manner by accounting for the plated volume and dilution factor. A linear relationship between log_10_(CFU mL^−1^) and log_10_ (dilution) with coefficient of determination R^2^ = 0.99337 supports the suitability of the device for rapid CFU estimation.

**Figure 10:**
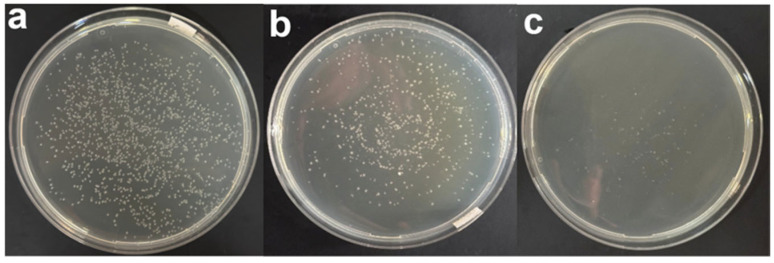
Dispersion of *E. coli* on LB agar plates using the handheld vibrational droplet generator. Representative plates inoculated with (**a**) undiluted culture, (**b**) 10-fold diluted culture and (**c**) 100-fold diluted culture. The device produces fine droplets over the plate surface, yielding well-separated colonies whose density decreases with increasing dilution, demonstrating uniform sample dispersion suitable for CFU enumeration.

Although these experiments are preliminary and based mainly on photographic evidence, they demonstrate that the vibrational droplet generator can indeed break up a bacterial suspension into many micro-droplets that land on the agar surface and grow into discrete, well-separated colonies. Figure 10 shows experiments with samples at different dilution concentrations.

## 4. Discussion

### 4.1. Advantages and Potential Applications

The present work shows that a simple, handheld, toothbrush-based device can produce reasonably monodisperse droplets and support both nucleic-acid amplification and microbial culture workflows. Key advantages include:

1. Accessibility and cost. All components are low-cost and widely available. This dramatically lowers the barrier to entry for labs that wish to explore droplet-based methods without investing in microfabrication or pump systems.
2. Simplicity of operation. Generating droplets is as simple as loading a pipette tip, mounting it on the adapter and turning on the toothbrush. No flow-rate tuning or complex tubing is required.
3. Flexibility of droplet size. By modifying only the pipette tip aperture and vibration amplitude, the same actuator can generate droplets suitable for digital assays (~100 µm) or for plate dispersion (~100–300 µm).
4. Compatibility with conventional workflows. Oil-based droplet emulsions can be imaged under standard microscopes, and sprayed droplets on LB plates integrate seamlessly with traditional CFU counting and colony picking procedures.

These features make the device attractive not only for resource-limited settings but also for educational labs, where students can directly see how mechanical vibration governs droplet formation and how digital assays work.

### 4.2. Future Applications

Although this work primarily focuses on demonstrating the performance of a toothbrush-vibration-driven handheld droplet generator for producing stable water-in-oil droplets, and on its proof-of-concept applications in digital LAMP and rapid CFU enumeration—highlighting the potential of such an ultra-minimal device in resource-limited settings—readers may also wish to understand how droplets generated by this front-end module could be interfaced with more mature microfluidic droplet-manipulation components to support more complex, multi-step analytical workflows. In practice, droplet microfluidics encompasses not only droplet generation but also downstream operations such as droplet splitting, merging, routing, trapping, and on-chip reagent addition, which are essential for building coherent and automated biochemical analysis pipelines.

In this context, Agnihotri et al. provided a systematic review of droplet splitting mechanisms in microfluidics, categorizing passive and active splitting strategies and analyzing how interfacial forces, flow conditions, and geometrical design work together to control volume partitioning and continuous operation [38]. This body of work offers both theoretical and practical guidance for implementing volume allocation and re-allocation once externally generated droplets are introduced into channel networks. Likewise, the review by Fergola et al. comprehensively covered droplet generation and manipulation strategies, including passive microchannel geometries, active actuation using electric/magnetic/acoustic fields, and downstream operations such as droplet trapping, immobilization, and sorting. These methods complement the upstream generation unit proposed here and provide a useful design framework for constructing an integrated “toolbox” for droplet-based microfluidic analysis [39].

In addition, reviews such as that by Xi et al. have summarized a range of active droplet-sorting techniques—including electric-, magnetic-, and acoustic-based approaches—that enable real-time selection and routing of droplets in microchannels based on internal signals or markers. Such capabilities are particularly important for multiplexed reaction pathways, sample selection, and closed-loop feedback workflows [40].

Building on these studies, one can envision using the toothbrush-driven generator as a general-purpose front-end module to produce droplets that are subsequently transferred into PDMS or thermoplastic microfluidic chip networks for downstream operations. For example:

- Splitting and recombination: By incorporating T- or Y-junction splitters and implementing passive/active splitting units described by Agnihotri et al., a single larger droplet could be divided into smaller sub-droplets to adjust reaction volume or effective concentration.
- Merging and reagent addition: Using droplet docking interfaces or connector structures, droplets from different sources could be merged within microchannels while additional reagents are introduced, enabling stepwise reactions or combinatorial screening.
- Routing and sorting: Electrically, magnetically, or acoustically actuated sorting modules could classify droplets based on internal reaction readouts (e.g., fluorescence intensity) and direct them to different outlets or detection zones, providing a foundation for high-throughput analyses and feedback control.

Such integration strategies would enable multi-step workflows (e.g., sequential reagent addition, serial dilution, and condition screening), more advanced digital assays (including preparation steps for sequencing or protein analysis), and high-throughput combinatorial screening. By coupling a toothbrush-based front-end droplet generator with standard downstream microfluidic modules, future work could establish an ultra-low-cost, user-friendly, and scalable droplet analysis platform that leverages the strengths of droplet microfluidics in bioanalysis, clinical diagnostics, and synthetic biology, while retaining the simplicity and field-deployability of handheld droplet generation.

### 4.3. Current Limitations

Several limitations of the present prototype should be acknowledged:

- Device-to-device and batch variability. Because pipette tip apertures are created manually by heat-shrinking around microneedles, aperture diameters vary between tips. This leads to differences in droplet size and sometimes in the onset of stable jetting. Similarly, minor differences in how the counterweight is attached can change the effective vibration amplitude. Producing standardised tips (e.g., moulded or laser-drilled) and a more reproducible counterweight mechanism would improve uniformity.
- Droplet motion and imaging challenges. Droplets are generated into an open Petri dish filled with oil. During and shortly after generation, continuous vibration causes droplets to move and occasionally float upwards or downwards in the depth of the dish. This motion complicates time-lapse imaging and quantitative tracking of individual droplets. In this work we mitigated the issue by stopping the vibration and allowing droplets to settle before imaging, but for live observation of reaction dynamics a shallower observation chamber or density-matched oil could be beneficial.
- Limited quantitative validation. The LAMP and *E. coli* plate experiments were deliberately designed as proof-of-concept demonstrations. Sample numbers are small, and there is little replication or comparison with established benchtop methods. While the observed trends are encouraging, rigorous analytical performance (limit of detection, accuracy, precision, robustness) has not yet been established.
- Lack of integrated temperature control. For nucleic-acid amplification, an external oven was used to maintain 65 °C. Integration of a simple heating module, or use of an existing incubator while retaining handheld droplet generation, would streamline workflows.

### 4.4. Broader Relevance to Biosensing and Single-Cell Heterogeneity

The integration of microfluidics with biosensing technologies offers substantial potential for next-generation lab-on-a-chip platforms and point-of-care (POC) diagnostics. Luka et al. (2015) reviewed how such integration combines biorecognition elements with microfluidic channels to reduce sample and reagent volumes, enhance mass transport and mixing, and consolidate sample preparation and detection within a single platform, thereby significantly improving sensitivity, real-time capability, and multiplexing potential [41]. This review also highlighted the diversity of continuous-flow, droplet-based, and digital microfluidic systems, as well as their prospective applications in precision agriculture, environmental monitoring, and clinical diagnostics.

Droplet-based digital analysis (e.g., ddPCR and ddLAMP) is one of the core applications driving this integrated trend. By partitioning a sample into a large number of small-volume compartments (such as droplets), digital assays enable statistical quantification at the single-target level, thereby markedly improving quantitative accuracy and dynamic range. Related work has shown that a portable system combining a vibrating sharp-tip capillary with digital isothermal amplification can generate tunable droplet sizes without external pumps or complex microfabricated structures, and can achieve digital nucleic-acid detection over a range of ~2 to ~6000 copies/µL [42]. Using a simple signal generator and a low-power vibration mechanism, this approach illustrates how low-cost droplet generators can be embedded into quantitative biosensing workflows and deliver high-dynamic-range digital amplification under resource-limited conditions.

Beyond nucleic-acid quantification, microfluidic systems have also been widely used for single-cell and heterogeneity analyses, revealing cell-to-cell behavioral differences that are masked by population-averaged measurements. For example, in reproductive biology, researchers have used droplet microfluidics to encapsulate individual sperm cells and track their dynamics in compartments of different sizes, thereby uncovering how microenvironmental constraints influence motility. Conceptually, this mirrors how droplet compartmentalization and amplification in digital assays isolate single target molecules or CFUs to reveal otherwise hidden functional heterogeneity.

### 4.5. Comparison with Portable and Chip-Free Droplet Generators

To more comprehensively situate this work within the landscape of existing portable and chip-free droplet generation technologies, it is necessary to compare key performance metrics, including droplet size range, throughput (droplet generation rate), system complexity, and dependence on external equipment or microfabricated chips. In He et al., a vibrating sharp-tip capillary approach leveraged acoustic streaming to enable efficient droplet generation without an external pressure source [36]. This system allowed real-time tuning of droplet diameters from ~6.77 µm to 661 µm within a single setup, and generated highly monodisperse droplets (CV < 4%) at rates up to ~5000 droplets/s. The actuation power was below 60 mW, and the overall device could be driven by a battery-powered, low-cost signal generator. While this method substantially outperforms the present toothbrush-driven device in throughput and size tunability, it still requires a bespoke piezoelectric actuator and precise alignment of the glass capillary tip, which increases experimental setup and tuning complexity.

Trossbach et al. reported a portable negative-pressure-driven droplet generator assembled entirely from commercial components, designed to mitigate the reliance of many microfluidic systems on custom fabrication and costly pump hardware [33]. Although this platform still depends on microfabricated channels and fluidic connections—and typically requires external control (e.g., a laptop) to regulate negative pressure—it demonstrated stable, size-tunable water-in-oil droplet generation within a chip, highlighting the design trade-offs between portability and system integration.

Earlier work by Chen et al. presented a handheld, power-free microfluidic droplet generator for applications such as single-cell genomic analysis and digital PCR, capable of producing monodisperse droplets [31]. While advantageous in cost and the absence of external power, this design still relies on microchannel chip structures and manual control, and thus remains more device-dependent than fully chip-free approaches.

By contrast, the toothbrush-driven droplet generator offers a distinctive advantage in that it integrates both the actuation mechanism and the droplet generation unit into a single consumer-grade device, eliminating the need for microfabricated chips, external pumps, dedicated signal generators, or complex alignment procedures. Under typical operating conditions, it can continuously generate droplets at an order-of-magnitude rate (e.g., ~100 droplets/s with CV ~3–5%). Although this throughput is lower than that of the vibrating-capillary system reported by He et al., the toothbrush-based approach offers unique benefits in experimental simplicity, ease of use, and accessibility. Compared with chip-dependent solutions (e.g., Trossbach and Chen), it avoids barriers associated with microstructure fabrication and chip-to-world interconnections, making it potentially more deployable in resource-limited settings and rapid field-testing scenarios.

For an intuitive comparison, Table 5 below provides a concise summary table:

### 4.6. Differences in Device Performance Discussion

Device-to-device variability is an inherent consideration for this prototype and was briefly noted in the Abstract and Conclusions; however, it was not discussed in sufficient detail in the main text. Here, “device-to-device variability” may arise from (i) differences among electric toothbrush units (even within the same model) and/or (ii) assembly-related variability when the nozzle/adapter is mounted and remounted on a given device. To preliminarily assess these effects, we tested multiple electric toothbrushes across different brands and models and characterized their vibration frequencies. While all devices operated within a broadly similar range (≈20,000–40,000 strokes per minute), we observed measurable differences in both frequency and amplitude, which can influence droplet size and generation rate. For instance, a Philips Sonicare toothbrush (sonic vibration, ~31,000 strokes per minute, ≈517 Hz) provided relatively stable droplet generation in our setup. Nonetheless, subtle unit-to-unit differences (e.g., motor performance and assembly tolerances) may still shift droplet formation behavior, underscoring the need for more systematic, quantitative evaluation of how such variability impacts performance.

## 5. Conclusions

We have developed and characterized a handheld vibrational droplet generator that repurposes a commercial electric toothbrush and disposable pipette tips to produce stable, nearly monodisperse water-in-oil droplets. Through a simple counterweight mechanism and manually tailored nozzle apertures, droplet diameters in the 100–300 µm range can be obtained at high generation rates without microfluidic chips or pumps.

Proof-of-concept experiments demonstrate that droplets produced by the device are compatible with isothermal nucleic-acid amplification and with microbiological colony formation. Digital LAMP assays using a feline parvovirus kit show the expected dependence of positive droplet fraction on template dilution, while *E. coli* plate experiments illustrate the device’s ability to act as a droplet-based spreader for CFU counting, replacing manual inoculation loops.

Although the current implementation still faces challenges in terms of device standardization, droplet motion in open dishes and limited quantitative validation, it already provides a simple, low-cost tool for digital assay exploration and microbiological sample dispersion. With modest further refinement and the additional reproducibility data outlined above, this approach could help democratize droplet-based technologies in both research and teaching laboratories and ultimately support portable “lab-in-hand” diagnostics in resource-limited environments.

## Figures and Tables

**Table 1:** Standard LAMP reaction composition (per 20 µL).

Reagent	Volume (µL)
LAMP Fluorescence Master Mix with UDG (2×)	10
LAMP Primer Mix (10×)	2
Bst DNA Polymerase	1
Positive Control (FPV DNA)	2
Nuclease-free water	5
Total	20

**Table 2:** Summarizes the droplet count statistics for the ~90 µm nozzle.

Number of Experiments	1	2	3	4
Times	5 s	10 s	15 s	20 s
Total	425	1120	1725	2260

**Table 3:** Representative droplet counts and positive fractions for FPV LAMP.

Number of Runs	Sample	Positive/Total	Positive Fraction *p* = *h*/*n*	λ	Relative Copy Numbers
Batch1	1× dilution	1848/1860	0.99	5.04	1
	5× dilution	1370/1760	0.78	1.51	0.2
	25× dilution	120/1710	0.08	0.07	0.04
Batch2	1× dilution	1776/1794	0.99	4.6	1
	5× dilution	1188/1606	0.74	1.35	0.2
	25× dilution	126/1638	0.08	0.08	0.04

**Table 4:** The colony count obtained at each dilution (*n* = 3).

Sample	First Run	Second Run	Third Run	Mean Colony Count	SD
1× dilution	1936	1872	2128	1978.67	132.23
10× dilution	328	376	416	373.33	44.06
100× dilution	32	28	44	34.67	8.33

**Table 5:** Comparison of different droplet generators.

Method	Droplet Size Range	Maximum Generation Rate	External Equipment Required	Reliance on Microfabricated Chips	Main Sources of Complexity
Toothbrush-driven droplet generator	~Variable (configuration-dependent)	~102 drops/s	Pump-/external power-free	No	Minimal setup; high ease of use
Vibrating sharp-tip capillary [36]	6.77–661 µm	≤5000 drops/s	Low-power signal generator/driver	No	Requires piezoelectric actuation and precise tip alignment
Negative-pressure microfluidics [33]	On-chip tunable	Moderate (pump-/geometry-dependent)	Requires pumps + computer control	Yes	Chip setup and fluidic
Handheld microchannel device [31]	On-chip monodisperse	Moderate	Manual operation	Yes	Dependence on manual pumping and microfluidic chips

## Data Availability

The original contributions presented in this study are included in the article/Supplementary Materials. Further inquiries can be directed to the corresponding author.

## Supplementary Materials

The following supporting information can be downloaded at: https://www.mdpi.com/article/10.3390/bios16010030/s1, Video S1: Droplet generated going into oil phase.
